# 2022 Acknowledgment of *mBio* Reviewers

**DOI:** 10.1128/mbio.03098-22

**Published:** 2022-12-20

**Authors:** Arturo Casadevall

**Affiliations:** a Johns Hopkins Bloomberg School of Public Health, Baltimore, Maryland, USA

## EDITORIAL

On behalf of the editors of *mBio*, I gratefully acknowledge the following members of our inaugural Junior Editorial Board who served as reviewers for the journal in 2022. The Junior Editorial Board began receiving scholarly publishing and peer review training in January, and formally began reviewing for *mBio* in September. These dedicated and well-qualified early-career researchers are a welcome addition to our community of reviewers.

Cynthia Adinortey
Daniel Aridgides
Lori Banks
Joseph Boll
Clotilde Bongrand
Jiandong Chen
Ousmane Cisse
Sebastien Crepin
Sophie Darch
Nicole De Nisco
Stephen Dolan
Elizabeth Draganova
Melissa Ellermann
Alexandre Gouzy
Melinda Grosser
Andrzej Harris
Tania Henríquez
Rebeccah Lijek
Rogério Lourenço
Bruno Martorelli Di Genova
Angela Mitchell
Roberto Molina-Quiroz
Irina Novikova
Francisca Nwaokorie
Ceren Ozkul
Atmika Paudel
Peter Ramirez
Lennart Schada von Borzyskowski
Anna Serquina
Liku Tezera
Zulema Udaondo Dominguez
Rachel Van Duyne
Daniel Velez-Ramirez
Julia Willett
Jason Yang
Zhengzhong Zou

On behalf of the editors of *mBio*, I gratefully acknowledge the following individuals, who served as reviewers for the journal in 2022. Their time and effort in reviewing articles have been essential in ensuring the high quality of our publications, and their help is greatly appreciated.

Robert B. Abramovitch
Sabrina Absalon
Michael C. Abt
Priyamvada Acharya
Abigail A. Adebusuyi
Sankar Adhya
Marie Adomako
Evelien M. Adriaenssens
Anton Aebischer
Herve Agaisse
Jon Agirre
Ellen Aho
Tuomas Aivelo
Caroline M. Ajo-Franklin
Intikhab Alam
Randy A. Albrecht
Bree Aldridge
Gajender Aleti
Helen Alexander
Irving Coy Allen
Francis Alonzo
Craig Altier
Shoshy Altuvia
Gaya Amarasinghe
Matthew Zack Anderson
Tim Anderson
Bjorn Andersson
David R. Andes
José M. Andrade
Mireille Ansaldi
Haike Antelmann
Rafael Araos
Chelsie Elizabeth Armbruster
Damien Arnoult
Irina Artsimovitch
Ann M. Arvin
Philip M. Ashton
David S. Askew
Claudia R. Avalos
Amirhesam Babajani
Brett M. Babin
Michael A. Bachman
Brian D. Badgley
Nicholas Baetge
Zeynep Baharoglu
Andy Bailey
Bill J. Baker
David A. Baker
Matthew G. Bakker
Siddharth Balachandran
Jose Luis Balcazar
Janneke Balk
Elizabeth Ballou
David A. Baltrus
Arinjay Banerjee
Arup Banerjee
Jillian F. Banfield
Fernando Baquero
Claudine Baraquet
Nazir Barekzi
John N. Barr
Alberto Bartesaghi
Richard Bartfai
Antonio Bartoletti
Alienor Baskevitch
Christopher F. Basler
Quique Bassat
Bonnie Bassler
Aninidya Basu
Joyoti Basu
Himanshu Batra
Aurelia Battesti
Tom J. Battin
Anthony D. Baughn
Özgür Bayram
William N. Beavers
Josh R. Beck
Anke Becker
Eric D. Becraft
Morgan Beeby
James G. Beeson
Sam Behar
Samantha L. Bell
George A. Belov
Alexia A. Belperron
Daniel Belstrøm
Ronen Ben-Ami
Jose A. Bengoechea
Mikkel Bentzon-Tilia
Mary Berbee
Roeland Lucas Berendsen
Stephen B. Beres
Julien Bergeron
Megan Bergkessel
Ben Berkhout
Francesco Berlanda Scorza
Thomas G. Bernhardt
Harris D. Bernstein
Sonja M. Best
Sébastien Besteiro
David Bhella
Dawn R. D. Bignell
Blake Billmyre
Jordan Bisanz
Heather N. Bischel
Wilbert Bitter
David F. Blair
Daniel Blanco-Melo
Matthew G. Blango
Joel N. Blankson
Michael Blatzer
Mike Blazanin
Tim R. Blower
Anamelia Lorenzetti Bocca
Paul L. Bodelier
James Boedicker
Carolyn H. Bohach
Jens Bohne
Tobias Bollenbach
Morgane Bomsel
Juan Bonachela
Ivo G. Boneca
Mariangela Bonizzoni
Khushboo Borah
Christophe Bord
Kathleen Boris-Lawrie
Katherine A. Borkovich
Jeffrey L. Bose
Michael Boshart
Alberto Bosque
Anne Botteaux
Sophie Bouillet
Nathalie Boulanger
Nicole M. Bouvier
Aoife Boyd
Jeffrey Michael Boyd
Zbynek Bozdech
Peter J. Bradley
L. Jeannine Brady
Alessandra Bragonzi
Axel A. Brakhage
Marc Bramkamp
Gerhard H. Braus
Jason M. Brenchley
John Briggs
Shaun R. Brinsmade
Robert A. Britton
Warwick Britton
Mathieu Brochet
Michael John Bromley
Roland Brosch
Alistair J. P. Brown
Daren W. Brown
Eric D. Brown
Kevin M. Brown
Edward P. Browne
Emily A. Bruce
John H. Brumell
Steven Bruner
Harald Brüssow
Mark P. Brynildsen
Bryan D. Bryson
Juliane Bubeck Wardenburg
Susan K. Buchanan
Angus Buckling
Nienke Buddelmeijer
Remi Buisson
Silvia Bulgheresi
Tione Buranda
Gaelen R. Burke
Briana M. Burton
Posy Busby
Carlos Andres Buscaglia
Kathryn Bushley
Geraldine Butler
Mark J. Buttner
Sarah Louise Caddy
Ken Cadwell
Melissa J. Caimano
Ben Callahan
Jodi L. Camberg
Richard D. Cannon
Douglas G. Capone
Rey Carabeo
Valerie Jean Carabetta
Nicholas H. Carbonetti
Jan E. Carette
Paul Carini
Rita Carsetti
Agostinho Carvalho
James E. Casanova
Clayton C. Caswell
Sergio Daniel Catz
Gary Cecchini
Eleonora Cella
Can Cenik
Chang-Jun Cha
Chrispin Chaguza
Jianmin Chai
Leslie Chan
Michael C. W. Chan
Xavier Charpentier
Subhadeep Chatterjee
Minu Chaudhuri
Liang Chen
Qi Chen
Sheng Chen
Xian-Ming Chen
Qin Cheng
Ziqiang Cheng
Ambrose L. Cheung
Pak-Hin Hinson Cheung
Peter Chien
Jae Sung Cho
Mallory J. Choudoir
Graham Christie
Peter J. Christie
Haiyan Chu
Andrea Cimarelli
Jan Claesen
Eric Clambey
Justin R. Clark
Donald M. Coen
Jörn Coers
Dani Cohen
Lisa Cole
Kathleen Collins
Rita R. Colwell
Alex A. Compton
James P. R. Connolly
Tyrrell Conway
Laura Cook
Andrea Cooper
Roland A. Cooper
Emily K. Cope
Isabelle Coppens
Brendan Cormack
Rebecca M. Corrigan
Luis M. Corrochano
Peggy A. Cotter
Alejandro Couce
Timothy L. Cover
Jeffery S. Cox
Robert Craigie
Robert A. Cramer
Sean Crosson
Jie Cui
Paul J. Cullen
Peter J. Cullen
Michael Cunliffe
Melanie T. Cushion
Huanqin Dai
Ankur B. Dalia
Remus Dame
Ajai A. Dandekar
Mary E. Davey
Bryan W. Davies
Dennis R. Dean
Daniela De Biase
Piet A. J. de Boer
Zeger Debyser
Patrick H. Degnan
Sam Degregori
Kirk W. Deitsch
Juan Carlos de la Torre
Nicholas R. De Lay
Frank R. DeLeo
Maurizio Del Poeta
Elora G. Demers
Natalia de Miguel
Erick Denamur
Ye Deng
Marianne De Paepe
William H. DePas
Emily Derbyshire
Mark Derbyshire
Hilde De Reuse
Sanjay A. Desai
Wanderley de Souza
Ulrich Desselberger
Corrella S. Detweiler
Savita Devi
Rory D. de Vries
Megan M. Dewdney
Timothy Jack de Wet
Floyd E. Dewhirst
Stephanie DeWitte-Orr
George C. diCenzo
Thomas Dick
Laura Dickson
Allison Louise Didychuk
Diego G. Diel
Fred Dietrich
Lars Dietrich
Bauke W. Dijkstra
Jimmy D. Dikeakos
Chen Ding
Meike Dittman
Dirk P. Dittmer
Maziar Divangahi
Ray Dixon
Julianne T. Djordjevic
Ulrich Dobrindt
Roberto Docampo
Christian Doerig
Yohei Doi
Stefano Donadio
Xiuzhu Dong
Kelly S. Doran
Marte S. Dragset
Roger Draheim
Shaynoor Dramsi
Karl Drlica
Purnima Dubey
Jaquelin P. Dudley
Antonie Dufour
Aurélien Dumètre
Anne K. Dunn
Gary M. Dunny
Jeffrey D. Dvorin
Jonathan Dworkin
Martin Egan
Patrick Eichenberger
Shokrollah Elahi
Johanna R. Elfenbein
Hiba El Hajj
Omar El-Halfawy
Marie A. Elliot
Sean Elliott
Cherilyn A. Elwell
Michael Emerman
David Emerson
Iuliana V. Ene
Susanne Erdmann
Marc Erhardt
Jorge C. Escalante-Semerena
Jacob D. Estes
Prahathees J. Eswara
Ronald Drew Etheridge
Elena Evguenieva-Hackenberg
Sarah Elisabeth Ewald
Franziska Faber
Robert Fagan
Christina Faherty
Huizhou Fan
Jun Fan
Maya A. Farha
Allison Fay
Michael J. Federle
Anthony R. Fehr
Mario F. Feldman
Carl Feng
Pinghui Feng
Youzhi Feng
Michael T. Ferdig
Jessie Fernandez
Ana Fernandez-Sesma
Sebastien Ferreira-Cerca
Paul D. Fey
David A. Fidock
Kenneth A. Fields
Henri-Pierre Fierobe
Andrés Finzi
Derek J. Fisher
Connor Fitzpatrick
Patrick Flaherty
Klas Flardh
Erik K. Flemington
Sarah Jane Flint
Stephanie Ann Flowers
Joanne L. Flynn
Alexis Fonseca
James Craig Forrest
Sarah M. Fortune
Jarrod R. Fortwendel
George E. Fox
David Fox III
Amelie Fradet-Turcotte
Maria E. Francia
Aswanth Francis
Dara W. Frank
Gad Frankel
Kurt Frederick
David N. Fredricks
Eric O. Freed
Dorte Frees
Ulrich H. Frey
Ville Friman
Shinsuke Fujiwara
Klaus Fütterer
Michaela Ulrike Gack
Christian Gaebler
Marta M. Gaglia
Tom Gallagher
Michael Y. Galperin
Michael G. Ganzle
Ning Gao
Brandon Lee Garcia
Leonor García-Bayona
Alam Garcia-Heredia
Nisha J. Garg
Brigitte Gasser
Kent Gates
Erin C. Gaynor
Ricardo Tostes Gazzinelli
Baoxue Ge
Barney Geddes
Thomas Geisbert
Caroline Attardo Genco
Kati Geszvain
Benjamin Gewurz
Partho Ghosh
John G. Gibbons
David P. Giedroc
Tim Gilberger
Stacey D. Gilk
Jason J. Gill
Michael S. Gilmore
Paul R. Gilson
Michael L. Ginger
Jennifer B. Glass
N. Louise Glass
Erin Gloag
Lucy Glover
Emile Gluck-Thaler
Emanuela Gobbi
Adam Godzik
Christine Goffinet
Jeremy Gold
Daniel E. Goldberg
Marcia Goldberg
Ivo Gomperts Boneca
Irene Gonzalez
Andrew L. Goodman
Laura Brunengraber Goodman
Uri Gophna
Alexander E. Gorbalenya
Stephen V. Gordon
Jed Gorlin
Heinrich Georg Gottlinger
Eva Gottwein
Friedrich Götz
Celia W. Goulding
Mark Goulian
Neil A. Gow
Anjali Gowripalan
Ian Grainge
Jeffrey A. Gralnick
Urs F. Greber
Abby Green
Chris Greening
Rob I. Griffiths
Elisabeth Grohmann
Gigi Kwik Gronvall
Samantha Gruenheid
Angelika Gründling
Pablo Guardado-Calvo
John Guatelli
Marc-Jan Gubbels
Suryaram Gummuluru
Ozan Gundogdu
John Gunn
Ju-Tao Guo
Jun Guo
Ravindra Kumar Gupta
Kirstin Gutekunst
Bart L. Haagmans
Sebastian Haas
Mike Hadfield
Maria Hadjifrangiskou
Denita Hadziabdic Guerry
Max M. Haggblom
Daisuke Hagiwara
Martin W. Hahn
Vanessa L. Hale
Ruth M. Hall
Larry J. Halverson
Trinity L. Hamilton
Tobin Hammer
Tom Hammond
Lynn E. Hancock
Meaghan H. Hancock
Allison Hansen
Blake M. Hanson
William R. Harcombe
Clare Harding
Jason B. Harris
Steven Harris
Tajie Harris
Stephen C. Harrison
Dennis Hartigan-O’Connor
Amy L. Hartman
Asma Hatoum-Aslan
Theodora Hatziionnou
Vasili Hauryliuk
Philippe Marcel Hauser
Zvi Hayouka
Bin He
Hongxuan He
Patrick Hearing
Anna C. Hearps
Nicholas S. Heaton
Lars Hederstedt
Lizbeth Hedstrom
Stephan Heeb
P. Scott Hefty
David E. Heinrichs
Robert A. Heinzen
Mark T. Heise
David Heisler
Emily Hemann
Andrew Henderson
Jeffrey P. Henderson
David R. Hendrixson
Sander Herfst
Christopher Hernandez
Martina Herrmann
Penelope Higgs
Yuta Hikichi
Russ Hille
Julian F. Hillyer
Deborah Hinton
Sarah M. Hird
Ya-Chi Ho
Anne G. Hoen
Dirk Hoffmeister
Tobias M. Hohl
Lone Høj
Matthew T. G. Holden
Erik F. Y. Hom
Thomas J. Hope
David Horn
Alexander R. Horswill
Walid A. Houry
Benjamin P. Howden
Andrew J. Hryckowian
Ansel Hsiao
Elaine Hsiao
Jianming Hu
Liya Hu
Kai Huang
Kerwyn Casey Huang
Lu Huang
Weishan Huang
Yao-Wei Huang
Elton Paul Hudson
Diego Huet
Diarmaid Hughes
Adam Joseph Hume
Peter Hunt
Christopher Hunter
Ryan C. Hunter
Julian G. Hurdle
James Hurley
Christopher Dwight Huston
Wilhelmina Huston
Michael F. Hynes
Joseph M. Hyser
Michael Ibba
Carolyn Ibberson
Cladia Igler
James Imlay
Roger W. Innes
Thomas R. Ioerger
Ronald M. Iorio
Nerea Irigoyen
Wael Ismail
Michael G. Ison
Solenne Ithurbide
Aravind Iyer
Lakshminarayan M. Iyer
Chandra Jack
Bethany Jackson
William R. Jacobs
Swati Jain
Tim James
Guilhem Janbon
James W. Janetka
Babak Javid
Grant J. Jensen
Paul R. Jensen
Byeonghwa Jeon
Travis Jewett
Zhongjun Jia
Cong Jiang
Shuo Jiao
Qusheng Jin
Jörgen Johansson
Eachan Johnson
Jarrod Johnson
Jeremiah G. Johnson
Marc C. Johnson
Kristina Jonas
Peter Jorth
Christine Josenhans
Sarah B. Joseph
Sebastian Joyce
Michael J. Joyner
Howard S. Judelson
Praveen Rao Juvvadi
David Kadosh
Barbara C. Kahl
Heimel Kai
Robert F. Kalejta
Markus Kalkum
Jana Kamanova
Nina Kamennaya
Jeremy Phillip Kamil
Jörg Kämper
Bavesh Davandra Kana
Cheng-Yuan Kao
Stefan H. I. Kappe
Melissa R. Kardish
Shahid Karim
Samuel Kariuki
Elizabeth Karr
Sophia Kathariou
Souichiro Kato
Louis Katz
Yoshihiro Kawaoka
Paul Kaye
Mary F. Kearney
Daniel B. Kearns
Thomas E. Kehl-Fie
Kylene Kehn-Hall
Kenneth C. Keiler
Nancy P. Keller
Eric Kemen
Linda J. Kenney
Scott P. Kenney
Stephen J. Kent
Christopher Kenyon
Nemat O. Keyhani
Nadeem Khan
Cezar M. Khursigara
Margaret Kielian
Minsu Kim
Sanggu Kim
Janine Kimpel
Christopher L. King
Frank Kirchhoff
Hannah L. Klein
Candice S. Klug
Leigh Knodler
Laura J. Knoll
Dennis C. Ko
Kazuo Kobayashi
Oren Kobiler
Theresa M. Koehler
Alan Koh
Andrew Young Koh
Julia Ruth Köhler
Ilana Kolodkin-Gal
James B. Konopka
Anna Konovalova
Kostas Konstantinidis
Michael Koomey
Daniel Kornitzer
Britt Koskella
Aleksandar D. Kostic
Vassili N. Kouvelis
Ákos T. Kovács
Lukasz Kozubowski
Karin E. Kram
Florian Krammer
Bernhard Krautler
Barry N. Kreiswirth
Tino Krell
Jens Kreth
Abby R. Kroken
Lee Kroos
Laurie T. Krug
Mart Krupovic
Damian J. Krysan
Richard J. Kuhn
Carol A. Kumamoto
Ayush Kumar
Purnima S. Kumar
Sanjai Kumar
Muthiah Kumaraswami
Barbara N. Kunkel
Markus Künzler
Kyung J. Kwon-Chung
Hyun Jin Kwun
Katherine Lagerstrom
Martin Lagging
Seema Lakdawala
Michael Lalk
Richard J. Lamont
Nathaniel Roy Landau
Andrew S. Lang
Stephanie N. Langel
Marc-Andre Langlois
Ryan A. Langlois
Robert A. Larossa
Thomas Ostenfeld Larsen
Amel Latifi
Gee W. Lau
Charles Lauhon
Adam S. Lauring
Maryse Lebrun
Benhur Lee
Soo Chan Lee
Adele Lehane
Jean-Jacques Letesson
Shoshana Levy
Gina R. Lewin
George Lewis
Janina P. Lewis
Kim Lewis
Zachary A. Lewis
Huiying Li
Jianrong Li
Kui Li
Shitao Li
Yize Li
Wenju Liang
Paul Licciardi
Mathias Lichterfeld
Samuel Light
David Lilley
Efrem S. Lim
Dominique H. Limoli
Huancai Lin
Jun Lin
Tao Lin
Steven E. Lindow
Roger G. Linington
John J. LiPuma
Dene R. Littler
Yael Litvak
Guanshu Liu
Haipeng Liu
Quan Liu
Tong-Bao Liu
Wanhong Liu
Xiang Liu
Xiaochuan Liu
Andy LiWang
María A. Llamas
Robert Lochhead
James Locke
Shawn R. Lockhart
Kristen L. Lokken-Toyli
Saife Niaz Lone
S. Wesley Long
Randy Longman
Daniel López
Purificación López-García
Benjamin Lopman
Guodong Lu
Ling Lu
Jeremy Luban
Stephan Ludwig
Sebastian Luecker
Micah A. Luftig
Peter Adrian Lund
Elaine Luo
Zhao-Qing Luo
Sara Lustigman
Joseph Lutkenhaus
Erika I. Lutter
Bing Ma
Vanessa M. Macias
Jason Mackenzie
Goedele Maertens
Tololupe Mafa-Attoye
Jacques Mahillon
Fabienne Malagnac
Michael H. Malim
Danielle Malo
Reet Mändar
Mark J. Mandel
Pierre Mandin
Riccardo Manganelli
Lisa E. Manhart
Alexander Mankin
William Margolin
Matthew Marsden
Benoît S. Marteyn
Matthias Marti
Luisa Martinez-Pomares
Vincent G. Martinson
Masashi Kanayama
Ruth Catherine Massey
Shinji Masuda
Greg Matlashewski
Kenneth Matreyek
Norihisa Matsuura
Yoshiharu Matsuura
Yu Matsuura
Wendy Maury
Despoina A. I. Mavridou
Karen L. Maxwell
Kerrie L. May
Andreas Mayer
Mark J. McBride
Shonna M. McBride
Beth A. McCormick
John P. McCutcheon
Quinn S. McFrederick
Jeffrey Scott McLean
Jason S. McLellan
Joel McManus
Alan McNally
Michael A. McVoy
Paul Mead
Francis Mégraud
Maliheh Mehrshad
Anand Mehta
Sahar Melamed
Gregory Melikian
Artemio Mendoza-Mendoza
Dorothy Menefee
Frances Mercer
Jason Mercer
Timothy C. Meredith
Tera Meru
Dennis W. Metzger
Wei Miao
Eleftherios Michailidis
Katarzyna Mickiewicz
Laura Mike
Alita A. Miller
Emily Miller
Virginia L. Miller
W. Allen Miller
Óscar Millet
Tohru Minamino
Chad Mire
Biswajit Mishra
Noboru Mizushima
Mahtab Moayeri
Edward S. Mocarski
Katarzyna Modrzynska
Jennifer F. Moffat
Lucy Moleleki
Isabella Moll
Mareike Moller
Vicente Monedero
Andrew J. Monteith
Christopher Montgomery
Cary Moody
Tae Seok Moon
Minoru Moriyama
Joachim Morschhäuser
Uffe Hasbro Mortensen
Joachim Mortensen
Gregory William Moseley
Ashlee Vivien Moses
William J. Moss
Serge Mostowy
Walther Mothes
Christopher Moxon
W. Scott Moye-Rowley
Patrick Moynihan
Oliver Mueller-Cajar
Laurence Mulard
Rolf Müller
Conrad W. Mullineaux
Matthew A. Mulvey
Ulrike G. Munderloh
Karl Münger
Sergio Munoz-Gomez
Carol A. Munro
Eain A. Murphy
Colin Murrell
Hannu Myllykallio
Carey D. Nadell
Thomas Naderer
László Nagy
Akiniro Nakamura
Naweed Isaak Naqvi
Franz Narberhaus
Aravind Natrajan
Sonia Navas-Martin
Dan Naylor
Daniel E. Neafsey
Stuart J. Neil
Martha I. Nelson
Donna M. Neumann
Olivier Neyrolles
Johan Neyts
Wai-Leung Ng
Dao Nguyen
Timothy J. Nice
Michael Niederweis
William C. Nierman
Justin R. Nodwell
Nicholas Noinaj
Daniela Nordzieke
Philip J. Norris
Joshua D. Nosanchuk
Particia Nuttall
Andrew Oberst
Nicky O’Boyle
Conor P. O’Byrne
Christine M. O’Connor
Nana Ofori-Anyinam
Kristen M. Ogden
Marco Rinaldo Oggioni
Peter O’Hare
Paulo Oliveira
Martin Olivier
Michal A. Olszewski
Teresa R. O’Meara
Anders Omsland
Robert C. Orchard
Carlos J. Orihuela
Mehmet A. Orman
William D. Orsi
Takashi Osanai
Fernando A. Osorio
Bohdan Ostash
Albert Osterhaus
Luis Ostrosky-Zeichner
George A. O’Toole
Karen M. Ottemann
Wayne Outten
Julie Overbaugh
Heather J. Painter
Leopoldo Palma
Guy H. Palmer
Tracy Palmer
Bernhard O. Palsson
Tao Pan
Samay Pande
Amit Kumar Pandey
Surya Prakash Pandey
John C. Panepinto
Nigel Paneth
Amanda R. Panfil
Nicolas Papon
Vincent Parissi
Petra Patakova
Shelley M. Payne
Simone Pecetta
Andrew Pekosz
Miguel A. Penalva
Ines C. Pereira
Rushika Perera
Michael H. Perlin
Stanley Perlman
Jessamyn Perlmutter
Lena Pernas
Andreas Peschel
Anushya Petchiappan
Brian Peters
Jason M. Peters
William A. Petri
Stefan Pfeiffer
Minh-Duy Phan
Laurent Philippot
William Picking
Joern Piel
Mark Pimentel
Stacey Piotrowski
Richard K. Plemper
Patrick Plésiat
Eric Murnane Poeschla
Joe Pogliano
Georg Pohnert
Alessandra Polissi
Mauricio H. Pontes
Francesca Ponziani
Leslie B. Poole
Leo Lit Man Poon
Michel-Robert Popoff
Katarzyna Potrykus
Christopher Power
Gabriele Pradel
Sarah Pacocha Preheim
Thomas Alan Premeaux
William Prinz
Maraike Probst
Jose L. Puente
Aaron Webster Puri
Liang Qiao
Hecong Qin
Yongli Qin
Jorge Fabián Quarleri
Robert Andrew Quinn
Mark Radosevich
Manuela Raffatellu
Tracy Lyn Raivio
Kumaran S. Ramamurthi
Giordano Rampioni
Richard E. Randall
Tara M. Randis
Didier Raoult
Chad A. Rappleye
David Rasko
Philip N. Rather
Adam J. Ratner
Christoph Ratzke
Isabella Rauch
Andrew Read
Johannes G. Rebelein
Manjula Reddy
Alan Rein
Olaya Rendueles Garcia
Laurent Renia
Michelle Lynne Reniere
Francis Repoila
Nicole Reynolds
Daniel D. Rhoads
Andrew Rice
Louis B. Rice
Dave Richard
Jonathan P. Richardson
Simone Richardson
Karen Ridge
Alain Rince
Michael D. Robek
Christopher M. Robinson
Keven Mara Robinson
César Rodríguez
Jerónimo Rodríguez-Beltrán
Luis Rodriguez-R
Roderich Roemhild
Paul D. Roepe
Geraint B. Rogers
Holger Rohde
Philippe Roingeard
Antonis Rokas
Jose Rolando
Pascale Romby
James C. Romero-Masters
Ute Römling
Davide Roncarati
Anthony Rongvaux
Regin Ronn
R. Martin Roop
German Rosas-Acosta
Jason W. Rosch
Miriam A. Rosenbaum
Brad R. Rosenberg
Maria Rosenthal
Rodney Rothstein
Jean-Pierre Routy
Christopher Ruis
Natividad Ruiz
Michael Rust
Joel L. Sachs
David L. Sacks
Jose Saenz
Saveez Saffarian
Olga Sakwinska
Claudia Sala
Maria-Carla Saleh
Alessandra Mattos Saliba
Antoine-Emmanuel Saliba
Jeanne Salje
Maria Sandkvist
Lamba Sangare
Dominique Sanglard
Sumana Sanyal
Ligia M. Saraiva
Peter Sarnow
Satish Raina
Jimmy H. Saw
Joy Scaria
Luis M. Schang
Birgit E. Scharf
Dirk-Jan Scheffers
Mario Schelhaas
Mark A. Schembri
Celia A. Schiffer
Larry S. Schlesinger
Susan Schlimpert
Jonas Schluter
Eric Schmidt
John L. Schmitz
Dirk Schnappinger
Dirk Schneider
William M. Schneider
John Schoggins
Klaus Schughart
Thomas F. Schulz
Petra Schwille
Guillaume Schwob
Barry Scott
Kathleen M. Scott
Michelle R. Scribner
Peter Sebo
Patrick R. Secor
Florian Seebeck
Anna Maria Seekatz
Michael Seidl
Anna Selmecki
Maria Selmer
Ranjan Sen
Keun Seok Seo
Laura R. Serbus
Alexander Serganov
David Serre
Peter Setlow
Karl Seydel
Mohammad R. Seyedsayamdost
William M. Shafer
Reut Shalgi
Stephanie R. Shames
Tongling Shan
Michael Shapira
Rebecca S. Shapiro
Anupam Sharma
Lokesh Sharma
Pushkar Sharma
Thomas J. Sharpton
Dana K. Shaw
Lindsey N. Shaw
Megan L. Shaw
Frederick Sheedy
Moshe Shemesh
Aimee Shen
Daniel Sher
Jianwei Shi
Pei-Yong Shi
Zheng-Li Shi
Zhou Jason Shi
Thomas M. Shinnick
Neta Shlezinger
Maya Shmulevitz
Erika Shor
Paul Sigala
Anita Sil
Robert F. Siliciano
Choon Kiat Sim
Augusto Simoes-Barbosa
Brent W. Simpson
Les Sims
Anthony P. Sinai
Steven Sinkins
Barbara S. Sixt
Eric P. Skaar
Rebecca L. Skalsky
Ute M. Skiba
Betty Slagle
James M. Slauch
Joan L. Slonczewski
Clyde A. Smith
Joe Smith
Kerry S. Smith
Evan S. Snitkin
Lori Snyder
Cecilia Söderberg-Nauclér
Greg A. Somerville
Hokyoung Son
Fernando C. Soncini
Nahum Sonenberg
Fengming Song
Jeongmin Song
Lihua Song
Harini Sooryanarain
Kevin R. Sowers
Jim Spain
Charles Specht
Alexander Speer
James Spencer
Melanie Spero
Tobias Spielmann
Alfred Michael Spormann
Rachel R. Spurbeck
Megan E. Spurgeon
Apollo Stacy
Jason E. Stajich
Nicola R. Stanley-Wall
Kenneth A. Stapleford
Chad Steele
Ann M. Stock
Giannis Stringlis
Xin-Zhuan Su
Agathe Subtil
William Sullivan
Michael Summers
Sheng Sun
Xianyun Sun
Margaret Sunde
Meera Surendran Nair
Troy C. Sutton
Sine Lo Svenningsen
Sarah Lauren Svensson
W. Edward Swords
Christine M. Szymanski
Taichi E. Takasuka
Hiroki Takeuchi
Kawsar Talaat
Rita Tamayo
Demeng Tan
Gene S. Tan
Peng Tan
Arif Mohammad Tanmoy
Gerald W. Tannock
Yosuke Tashiro
Gregory Taylor
Nicholas Taylor
Paulo José Pereira Lima Teixeira
Michaela A. Teravest
Blanka Tesla
Megan Thoemmes
David L. Thomas
Paul G. Thomas
Vinai Chittezham Thomas
Kai M. Thormann
Dagang Tian
James M. Tiedje
Mariana Tinajero-Trejo
Dirk Tischler
Aaron A. R. Tobian
David M. Tobin
Jonathan D. Todd
Gaku Tokuda
Fuyuki Tokumasu
Martin Tolstrup
Stephen M. Tompkins
Yaojun Tong
Chris J. Tonkin
Masanori Toyofuku
Paula Traktman
Nicolas Oswaldo Trinler
Chi-Ching Tsang
Tommy Tsan-Yuk Lam
Victor Tse
Renee M. Tsolis
Takafumi Tsuboi
Moriya Tsuji
Kyoko Tsukiyama-Kohara
Ayako Tsushima
Kürsad Turgay
Anne-Marie Turner
Rodney K. Tweten
William Tyor
Anne-Catrin Uhlmann
David M. Underhill
Stephan Uphoff
Stéphane Uroz
Daniel Utter
Balázs Vajna
Vito Valiante
Marcelo A. Vallim
Daan M. van Aalten
Charles Van der Henst
Brian C. VanderVen
Marjan W. van der Woude
Stineke van Houte
Debby van Riel
Julien Varaldi
Arvind Varsani
Andres Vazquez-Torres
Anthony G. Vecchiarelli
Alain Veilleux
Stephanus N. Venter
Jorge E. Vidal
Manigandan Lejeune Virapin
Karen L. Visick
Anastasia N. Vlasova
Joseph Vogel
Uwe Völker
Tobias Vonnahme
Julia A. Vorholt
Joseph Thomas Wade
Navish Wadhwa
Johannes Wagener
Kshitij Wagh
Nicholas Waglechner
Matthew K. Waldor
Kevin John Waldron
Edward Walker
Daniel Wall
Nicholas A. Wallace
Jingwen Wang
Shanquan Wang
Huizhi Wang
Jue D. Wang
Linqi Wang
Ping Wang
Yiping Wang
Martin J. Warren
Kenneth Wasmund
Robert O. Watson
Kylie J. Watts
Richard J. Webby
Mary Weber
Roland Wedlich-Soeldner
James Weger-Lucarelli
Aaron Weimann
Brian C. Weinrick
Virginia Weis
Carol D. Weiss
David S. Weiss
Howie Weiss
Susan R. Weiss
Laure Weisskopf
Martin Welker
Zezhang Tom Wen
Tim Wencewicz
Guido Werner
A. Phillip West
Nicholas P. West
Kelsey Wheeler
Fiona J. Whelan
Sean P. J. Whelan
Elizabeth A. White
Judith M. White
Kris White
Philip Lewis White
Aaron Whiteley
Katrine Whiteson
Malcolm Whiteway
John C. Whitney
Nathan P. Wiederhold
Annegret Wilde
Robert J. Wilkinson
Ronnie G. Willaert
Rob J. L. Willems
John V. Williams
Paul Williams
Kim C. Williamson
Anusuya Willis
Mark R. Wills
Daniel N. Wilson
Richard A. Wilson
Shelby Winans
Wade C. Winkler
Elizabeth Winzeler
Christopher Witzany
Harald Wodrich
Christiane Wolz
Lok-Yin Roy Wong
Richard Wong
Thomas K. Wood
Christopher W. Woods
Joshua J. Woodward
Rachel A. F. Wozniak
Brendan W. Wren
Chao Wu
Qingfa Wu
Ruonan Wu
Sebastian Thomas Wurster
Tingting Xiang
Yan Xiang
Deng Xin
Changsheng Xing
Chaoyang Xue
Gilad Yakov
Masahiro Yamamoto
Aixin Yan
Xia Yan
Ence Yang
Liang Yang
Wan Yang
George S. Yap
Felix Yarovinsky
Wenwu Ye
Hui-Ling Yen
Jianyi Yin
Kristine Yoder
Avihu Yona
Hyunah Yoon
Sukhwan Yoon
Souheil Younes
Peter Young
Nicholas D. Youngblut
Nathan Yozwiak
Huibin Yu
Jae-Hyuk Yu
Yunsong Yu
Zhenzhong Yu
Hanna S. Yuan
Jing Yuan
Lijuan Yuan
Kwok-Yung Yuen
Allan Zajac
Hasan Zaki
Sarah Zanders
Qinglu Zeng
Quan Zeng
Fushun Zhang
Haifeng Zhang
Weiwen Zhang
Yu Zhang
Zhengguang Zhang
Jincun Zhao
Qingfei Zheng
Yong-Hui Zheng
Hongbo Zhou
Xiaohui Zhou
Fanxiu Zhu
Lixin Zhu
Jochen Zimmer
Peter A. Zimmerman

